# Photoreceptor Disc Enclosure Occurs in the Absence of Normal Peripherin-2/rds Oligomerization

**DOI:** 10.3389/fncel.2020.00092

**Published:** 2020-04-28

**Authors:** Tylor R. Lewis, Mustafa S. Makia, Mashal Kakakhel, Muayyad R. Al-Ubaidi, Vadim Y. Arshavsky, Muna I. Naash

**Affiliations:** ^1^Department of Ophthalmology, Duke University Medical Center, Durham, NC, United States; ^2^Department of Biomedical Engineering, University of Houston, Houston, TX, United States; ^3^College of Optometry, University of Houston, Houston, TX, United States; ^4^Department of Pharmacology and Cancer Biology, Duke University Medical Center, Durham, NC, United States

**Keywords:** photoreceptor, peripherin, outer segment, disc, retina

## Abstract

Mutations in the peripherin-2 gene (*PRPH2*, also known as *rds*) cause a heterogeneous range of autosomal dominant retinal diseases. *PRPH2* encodes a photoreceptor-specific tetraspanin protein, PRPH2, that is a main structural component of the photoreceptor outer segment. PRPH2 distributes to the rims of outer segment disc membranes as they undergo the process of disc membrane enclosure. Within these rims, PRPH2 exists in homo-oligomeric form or as a hetero-oligomer with another tetraspanin protein, ROM1. While complete loss of PRPH2 prevents photoreceptor outer segment formation, mutations affecting the state of its oligomerization, including C150S, C213Y and Y141C, produce outer segment structural defects. In this study, we addressed whether any of these mutations also affect disc enclosure. We employed recently developed methodology for ultrastructural analysis of the retina, involving tissue processing with tannic acid, to assess the status of disc enclosure in knockin mouse models bearing either one or two alleles of the C150S, C213Y and Y141C PRPH2 mutations. While varying degrees of outer segment structural abnormalities were observed in each of these mouse models, they contained both newly forming “open” discs and mature “enclosed” discs. These data demonstrate that normal PRPH2 oligomerization is not essential for photoreceptor disc enclosure.

## Introduction

According to the Human Gene Mutation Database (Stenson et al., 2014), there are over 190 mutations in the peripherin-2 gene, *PRPH2*, that cause a heterogeneous set of retinal dystrophies, including retinitis pigmentosa, pattern dystrophy and macular dystrophy (Boon et al., 2008). *PRPH2* encodes the photoreceptor-specific tetraspanin protein, PRPH2, that resides within the photoreceptor outer segment, a specialized light-sensitive ciliary organelle containing a stack of disc-shaped membranes, or “discs,” that harbor the molecular machinery performing phototransduction.

**Figure 1:**
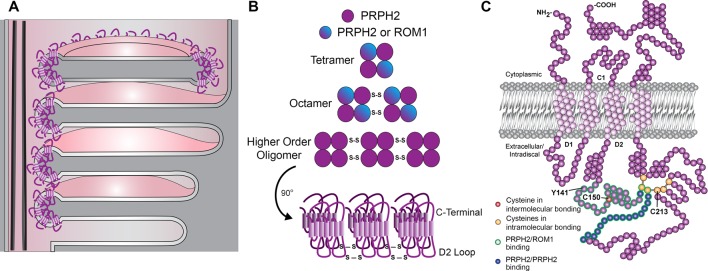
Schematic representations of outer segment disc enclosure, peripherin-2 gene (PRPH2) oligomerization and PRPH2 molecular structure.** (A)** The base of a rod photoreceptor outer segment contains both “open,” nascent discs that are exposed to the extracellular space and “enclosed,” mature discs that are separated from the outer segment plasma membrane. In open discs, PRPH2 is located only at the discs rims adjacent to the connecting cilium. In enclosed discs, PRPH2 is located throughout the entire circumference of the disc. **(B)** The formation of PRPH2/ROM1 homo- and hetero-oligomers involves disulfide bonds between cysteines within the D2 loop of each molecule. **(C)** The molecular structure of PRPH2 is depicted with several features highlighted. Of note, the D2 loop contains seven cysteines: the C150 residue that is involved in intermolecular bonds for oligomerization and six that are involved in intramolecular bonds, including C213. This loop also contains the Y141 residue. Modified from Stuck et al. (2016) with permission by Elsevier.

Photoreceptor discs are formed through serial evagination of the outer segment plasma membrane (Steinberg et al., 1980; Burgoyne et al., 2015; Ding et al., 2015; Volland et al., 2015) mediated by actin polymerization (Chaitin et al., 1984; Williams et al., 1988; Boitet et al., 2019; Spencer et al., 2019b). In newly evaginating discs, PRPH2 is concentrated at the membrane adjacent to the ciliary axoneme; as growing discs reach their final diameter, they undergo the process of enclosure within the outer segment (complete in rods or partial in cones), with PRPH2 redistributing throughout their rims (Figure 1A; Arikawa et al., 1992; Ding et al., 2015; Stuck et al., 2016). While PRPH2 redistribution is associated with disc enclosure, the exact mechanism underlying this complex membrane rearrangement remains unknown. Notably, the lack of PRPH2 in the *retinal degeneration slow* (*rds*) mouse completely prevents photoreceptor disc formation and is accompanied with a massive release of extracellular vesicles, or ectosomes, from the photoreceptor cilium (Cohen, 1983; Jansen and Sanyal, 1984; Nir and Papermaster, 1986; Usukura and Bok, 1987; Chakraborty et al., 2014; Salinas et al., 2017).

Given the essential role of PRPH2 in maintaining the photoreceptor outer segment structure, much effort has focused on its molecular and supramolecular organization (Figures 1B,C). In photoreceptors, PRPH2 exists as either a homo- or a hetero-tetramer with rod outer segment membrane protein 1 (ROM1), another tetraspanin located at the disc rims (Loewen and Molday, 2000; reviewed in Stuck et al., 2016). These core tetramers can undergo covalent disulfide linkage to form homo- or hetero-octamers, with PRPH2 homo-octamers able to form even higher-order oligomers connected by additional disulfide bonds (Loewen and Molday, 2000; Chakraborty et al., 2009, 2010; Zulliger et al., 2018).

The covalent disulfide linkage involved in PRPH2 oligomerization occurs in the large intradiscal loop known as the D2 loop (Figures 1B,C and Goldberg et al., 1998; Kedzierski et al., 1999). Notably, the majority of known human mutations in *PRPH2* occur in the D2 loop (Boon et al., 2008), suggesting the functional significance of PRPH2 oligomerization. This loop contains seven cysteines (C150, C165, C166, C213, C214, C222 and C250) that could potentially form disulfide bonds (Figure 1C). In particular, mutation of C150 was the first shown to inhibit PRPH2 oligomerization *in vitro* (Goldberg et al., 1998; Loewen and Molday, 2000). Recent work has focused on the generation of mouse models to address the involvement of these cysteines in PRPH2 oligomerization and function. Three of these models were used in the present study.

Our first knockin mouse model contained the C150S mutation previously shown to inhibit the ability of PRPH2 to form higher-order oligomers and to disrupt outer segment structure (Chakraborty et al., 2009, 2010; Zulliger et al., 2018). Our second model contained the C213Y mutation found in patients with dominant pattern dystrophy (Zhang et al., 2002). This mutation also inhibits PRPH2 oligomerization while disrupting the outer segment structure in knockin mice (Chakraborty et al., 2020). The third, reciprocal model expressed the dominant disease-associated mutation, Y141C (Khani et al., 2003; Francis et al., 2005; Moshfeghi et al., 2006; Vaclavik et al., 2012). This mutation leads to an abnormal increase in the content of higher-order PRPH2 oligomers due to the formation of additional, ectopic disulfide bonds (Stuck et al., 2014; Conley et al., 2017). The abnormal increase in PRPH2 oligomerization in this mouse also disrupts the outer segment structure (Stuck et al., 2014; Conley et al., 2017). Yet, specific mechanisms connecting abnormal PRPH2 oligomerization with defects in the outer segment structure remain unknown. The possibility that PRPH2 oligomerization is essential for disc enclosure was addressed in the current study.

Historically, the ability to analyze disc enclosure, particularly in mutant, structurally disrupted outer segments has been notoriously difficult. The most direct approach is to reconstruct membrane architecture using three-dimensional electron tomography (Burgoyne et al., 2015; Volland et al., 2015). However, this technique is a resource- and labor-intensive and may not be suitable for analyzing mutant outer segments. Alternatively, newly forming discs exposed to the extracellular space could be distinguished from mature, fully enclosed discs in live tissues through their differential labeling by membrane-impermeable compounds, such as Procion or Lucifer yellow (Laties et al., 1976; Matsumoto and Besharse, 1985). However, this technique has not been adapted for mammalian retinas or unfixed tissue. A more recent, conceptually similar approach employs tannic acid as a contrasting agent for transmission electron microscopy (TEM; Ding et al., 2015). As tannic acid poorly penetrates intact membranes, it preferentially stains the membranes of newly forming “open” discs rather than fully matured enclosed discs. Importantly, this method applies to analyze mutant outer segments with structural defects of varying severity (Spencer et al., 2019a,b). Therefore, we employed the tannic acid staining protocol to analyze whether disc enclosure is affected in knockin mouse models bearing either one or two of the C213Y, Y141C and C150S PRPH2 mutations, which modulate its oligomerization. While the outer segment structure is perturbed in each of these mutants, we observed both darkly stained open discs and lightly stained enclosed discs, indicating that normal PRPH2 oligomerization is not necessary for photoreceptor disc enclosure.

## Materials and Methods

### Animals

Animal maintenance and experiments were approved by the local Institutional Animal Care and Use Committee (IACUC; University of Houston, Houston, TX, USA) and guidelines as stated by the Association for Research in Vision and Ophthalmology (Rockville, MD, USA). The generation of the C150S, C213Y and Y141C knock-in mice was previously described in Stuck et al. (2014), Zulliger et al. (2018) and Chakraborty et al. (2020), respectively. The *rds* mouse was generously provided by Neeraj Agarwal, Ph.D. (University of North Texas Health Science Center, Fort Worth, TX, USA). All mice were genotyped to ensure that they did not contain either the *rd8* (Mattapallil et al., 2012) or *rd1* (Pittler et al., 1993) mutations commonly found in inbred mouse strains. All mice were housed under a 12/12 h diurnal light (~30 lux) cycle.

### Transmission Electron Microscopy

Fixation and processing of mouse eyes for TEM was performed as described previously (Ding et al., 2015). Anesthetized mice were transcardially perfused with 2% paraformaldehyde, 2% glutaraldehyde and 0.05% calcium chloride in 50 mM MOPS (pH 7.4) resulting in exsanguination. Enucleated eyes were fixed for an additional 2 h in the same fixation solution at room temperature. Eyecups were dissected from fixed eyes, embedded in 2.5% low-melt agarose (Precisionary, Greenville, NC, USA) and cut into 200 μm thick slices on a Vibratome (VT1200S; Leica, Buffalo Grove, IL, USA). Agarose sections were stained with 1% tannic acid (Electron Microscopy Sciences, Hatfield, PA, USA) and 1% uranyl acetate (Electron Microscopy Sciences), gradually dehydrated with ethanol and infiltrated and embedded in Spurr’s resin (Electron Microscopy Sciences). Seventy nanometer sections were cut, placed on copper grids and counterstained with 2% uranyl acetate and 3.5% lead citrate (19314; Ted Pella, Redding, CA, USA). The samples were imaged on a JEM-1400 electron microscope (JEOL, Peabody, MA, USA) at 60 kV with a digital camera (Orius; Gatan, Pleasanton, CA, USA). Image analysis and processing were performed with ImageJ. For each genotype, over 100 outer segments from at least two mice of randomized sex were analyzed.

## Results

Because PRPH2 mutations may affect the outer segment content of this protein (Stuck et al., 2014; Zulliger et al., 2018; Chakraborty et al., 2020), we first addressed whether disc enclosure could be affected by reduced expression of PRPH2. This was performed using a heterozygous *rds* mouse (*rds*/+). Because the *rds* allele is essentially knockout (van Nie et al., 1978; Connell et al., 1991; Travis et al., 1991), this mouse has approximately one half of the normal PRPH2 content (Cheng et al., 1997) and displays defects in the outer segment structure, including the formation of large membrane “whorls” (Hawkins et al., 1985; Sanyal et al., 1986; Chakraborty et al., 2014).

**Figure 2:**
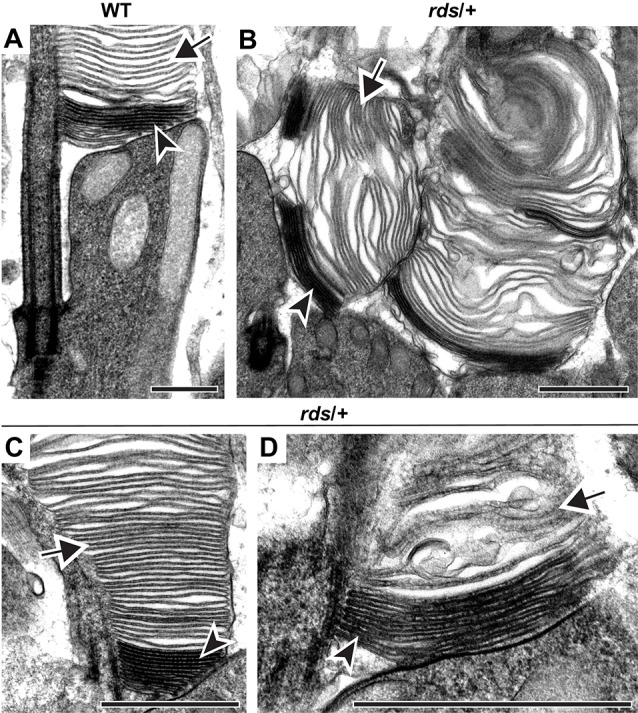
Reduced expression of PRPH2 disrupts outer segment structure without preventing disc enclosure. Transmission electron microscopy (TEM) of photoreceptor outer segments from 12-day old WT **(A)** or *rds*/+ **(B–D)** mice stained with tannic acid. For each genotype, over 100 different outer segments from three mice were analyzed. For all images, arrowheads mark darkly stained open discs while arrows mark lightly stained enclosed discs. Scale bars are 1 μm.

Tannic acid staining of *rds*/+ and WT rods are compared in Figure 2. In WT rods, several nascent discs evaginating at the outer segment base are stained more intensely than the mature, enclosed discs (arrowhead and arrow, respectively, in Figure 2A). Also, enclosed discs are slightly swollen compared to open discs, apparently due to osmotic fluctuations during tissue processing (see more examples in Ding et al., 2015; Spencer et al., 2019a,b). As previously reported, *rds*/+ rods displayed an array of outer segment abnormalities, however, they contained both darkly stained, nascent and lightly stained, mature discs (arrowheads and arrows, respectively, in Figures 2B–D). These data indicate that, while reduced expression of PRPH2 causes structural defects in outer segments, disc enclosure still occurs.

**Figure 3:**
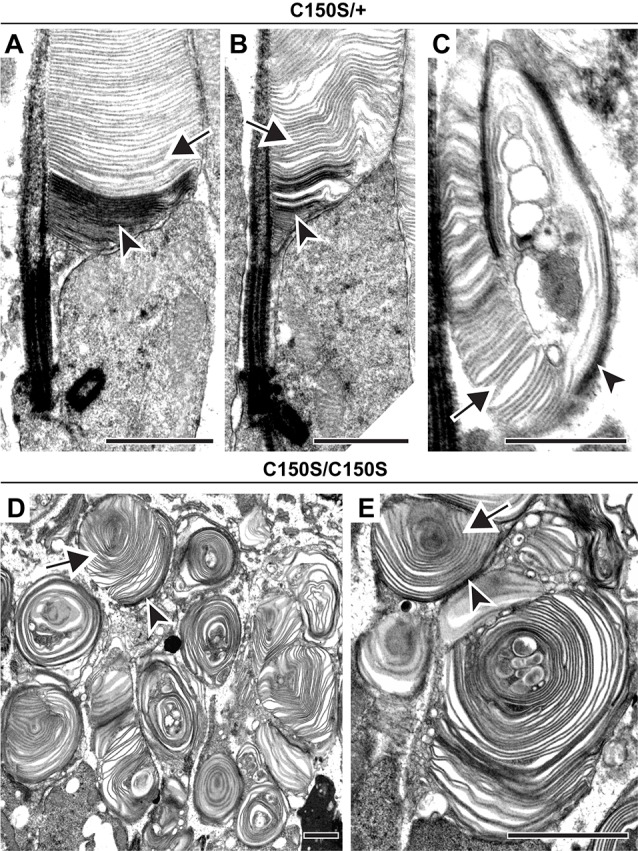
Expression of C150S PRPH2 disrupts outer segment structure without preventing disc enclosure.** (A–C)** TEM of photoreceptor outer segments of 30-day old heterozygous C150S (C150S/+) mice stained with tannic acid. **(D,E)** TEM of photoreceptor outer segments of 16-day old C150S homozygous (C150S/C150S) mice stained with tannic acid. For each genotype, over 100 different outer segments from two mice were analyzed. For all images, arrowheads mark darkly stained open discs while arrows mark lightly stained enclosed discs. Scale bars are 1 μm.

Next, we addressed whether disc enclosure is affected by abnormal PRPH2 oligomerization. We first analyzed the C150S knockin mouse in which oligomerization is inhibited (Zulliger et al., 2018). The ultrastructural analysis showed a diverse range of phenotypes. Heterozygous C150S animals contained both relatively normal outer segments and those with severely disrupted overgrown membrane structure, sometimes with vesicular material trapped inside (Figures 3A–C). Yet, no matter the severity of the structural phenotype, we always observed both darkly stained open and lightly stained enclosed discs (arrowheads and arrows, respectively, in Figures 3A–C). The outer segment structural abnormalities in homozygous C150S animals were much more significant, with nearly all outer segments shaped as membrane “whorls” (Figures 3D,E). Surprisingly, the majority of membranes comprising these whorls were still enclosed (arrow, Figures 3D,E). Therefore, at least in the context of the C150S mutation, normal PRPH2 oligomerization is not required for disc membrane enclosure.

**Figure 4:**
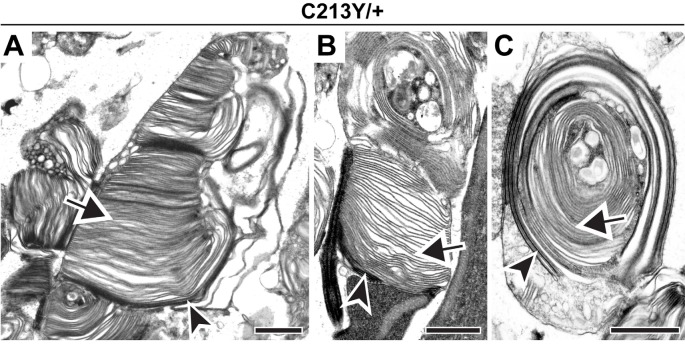
Expression of C213Y PRPH2 disrupts outer segment structure without preventing disc enclosure.** (A–C)** TEM of photoreceptor outer segments of 30-day old heterozygous C213Y (C213Y/+) mice stained with tannic acid. Over 100 different outer segments from two mice were analyzed. For all images, arrowheads mark darkly stained open discs while arrows mark lightly stained enclosed discs. Scale bars are 1 μm.

To further support this conclusion, we analyzed the C213Y knock-in mouse, another model in which PRPH2 oligomerization is inhibited (Chakraborty et al., 2020). Like in the C150S mutant, we observed an array of outer segment structural abnormalities in C213Y heterozygotes, with some cells producing distorted outer segments and others producing whorls (Figures 4A–C). Yet, in every case, we observed the distinct tannic acid staining pattern consistent with the majority of membrane structures being enclosed (arrows in Figures 4A–C). We also attempted to investigate outer segment structure in C213Y homozygotes, but observed a phenotype almost as severe as in the homozygous *rds* mouse, with no significant membrane elaborations emanating from the photoreceptor cilium (essentially as reported in Chakraborty et al., 2020).

**Figure 5:**
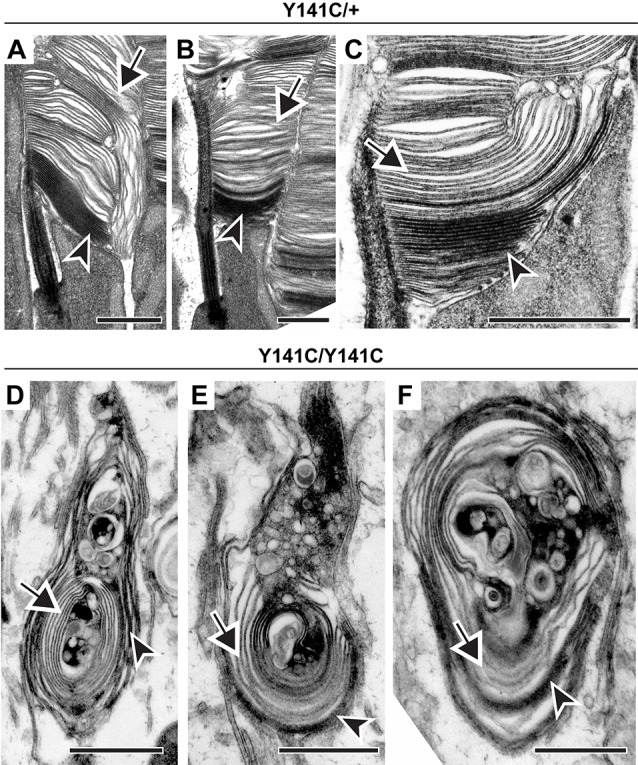
Expression of Y141C PRPH2 disrupts outer segment structure without preventing disc enclosure.** (A–C)** TEM of photoreceptor outer segments of 90-day old heterozygous Y141C (Y141C/+) mice stained with tannic acid. **(D–F)** TEM of photoreceptor outer segments of 16-day old Y141C homozygous (Y141C/Y141C) mice stained with tannic acid. For each genotype, over 100 different outer segments from two mice were analyzed. For all images, arrowheads mark darkly stained open discs while arrows mark lightly stained enclosed discs. Scale bars are 1 μm.

In the next set of experiments, we addressed whether the process of disc enclosure is affected by an abnormal increase in PRPH2 oligomerization in the Y141C knock-in mouse (Stuck et al., 2014; Conley et al., 2017). Similar to the C150S mutant, outer segments of heterozygous Y141C mice displayed an array of defects (Figures 5A–C). Again, despite these structural abnormalities, each outer segment contained both darkly stained and lightly stained discs (arrowheads and arrows, respectively, in Figures 5A–C). The outer segment structure of homozygous Y141C mice was significantly more distorted than in any other models analyzed here, with vesicle accumulation inside the outer segment being particularly prominent (Figures 5D–F). However, even within these structures, many membrane structures were more lightly stained and appeared swollen, indicating that they are enclosed (arrows in Figures 5D–F). Overall, this analysis suggests that an abnormal increase in PRPH2 oligomerization does not restrict disc enclosure from occurring.

**Figure 6:**
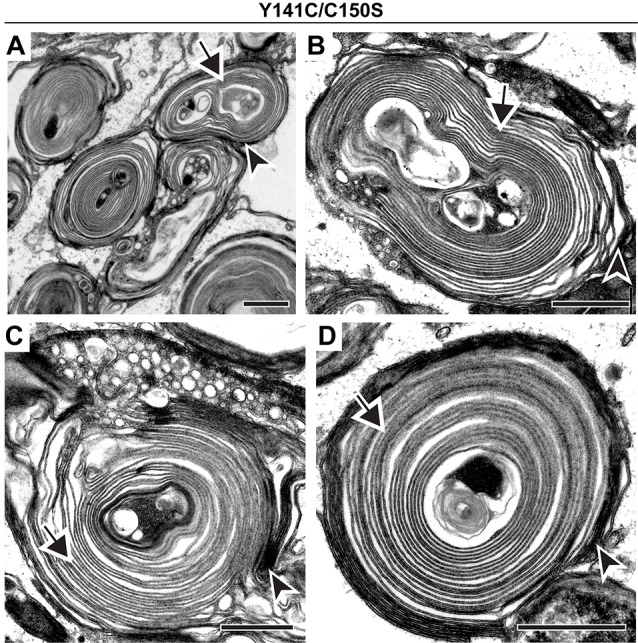
Co-expression of Y141C and C150S PRPH2 disrupts outer segment structure without preventing disc enclosure.** (A–D)** TEM of photoreceptor outer segments of 30-day old compound heterozygous Y141C/C150S mice stained with tannic acid. For each genotype, over 100 different outer segments from two mice were analyzed. For all images, arrowheads mark darkly stained open discs while arrows mark lightly stained enclosed discs. Scale bars are 1 μm.

Finally, we analyzed a compound mutant animal (Y141C/C150S) that expressed one copy of PRPH2 that inhibited and another that promoted PRPH2 oligomerization. A previous study (Zulliger et al., 2018) showed that this mutant is characterized by an overall increase in the amount of large PRPH2 oligomers (such as in the Y141C mutant alone) combined with a lack of PRPH2 dimers (such as in the C150S mutant alone). Our analysis of Y141C/C150S photoreceptors confirmed the extremely dysmorphic outer segment structure, whereby nearly all outer segments look like whorls (Figures 6A–D). Despite such profound membrane disorganization, each of these outer segments showed the usual pattern of darkly stained, open discs and lightly stained, enclosed discs (arrowheads and arrows, respectively, in Figures 6A–D). Thus, disc enclosure still takes place even with the complex perturbation of PRPH2 oligomerization in the Y141C/C150S mouse.

## Discussion

In this study, we analyzed three knock-in mouse lines (C150S, C213Y and Y141C) that exhibit either inhibition of or an abnormal increase in PRPH2 oligomerization (Stuck et al., 2014; Zulliger et al., 2018; Chakraborty et al., 2020). Remarkably, neither these nor the compound C150S/Y141C mutations prevented the process of photoreceptor disc enclosure despite causing various degrees of outer segment structural abnormalities. These data demonstrate that the enclosure of disc membranes is a very robust process, which can proceed even in the absence of precise disc alignment. This conclusion is corroborated by a recent report describing a mechanistically unrelated mouse model (the Arp2/3 knockout), in which outer segment morphogenesis is impaired due to inhibition of actin polymerization at the disc formation site (Spencer et al., 2019b). Photoreceptors of Arp2/3 knockout mice produce large whorl-like membranous structures instead of normal outer segments, yet the majority of membranes within these structures are enclosed.

If disc enclosure is unaffected by the mutations analyzed in our study, how do they cause such dramatic outer segment abnormalities? The most straightforward explanation is that these structural abnormalities are a direct consequence of abnormal PRPH2 oligomerization, which has been suggested previously (Conley et al., 2019). In that study, a chimeric protein consisting of the PRPH2 C-terminus attached to the tetraspanin core of ROM1 was able to initiate the building of disc membranes (as previously shown for other PRPH2 C-terminus chimeras; Salinas et al., 2017), but the maturation of outer segment structure, including the hairpin-like structure of disc rims, required the normal formation of PRPH2 oligomers. Another recent study showed that higher-order oligomers of PRPH2 are required for maintaining the continuity of disc rims (Milstein et al., 2020). In their study, transgenic expression of C150S PRPH2 in WT frogs (that have normal endogenous PRPH2 expression) led to the formation of ectopic incisures and disc rims, with the former being noted when these frogs were first generated (Loewen et al., 2003). Milstein et al. (2020) proposed that PRPH2 oligomers extend laterally along the circumference of the disc to maintain the continuity of the disc rim and to regulate incisure formation. They further suggested that defects in disc rim continuity (such as upon expression of C150S PRPH2) lead to the overall disruption in outer segment structure. The outer segment structure in the mouse mutants analyzed in the present study was too dysmorphic to assess disc rim continuity or the frequency of incisures. But even if such defects were present, our results indicate that they did not preclude disc enclosure. This may further suggest that PRPH2 may not be the primary factor responsible for the process of disc enclosure, but rather it localizes to the disc rim following the enclosure process.

We can offer two alternative explanations of the outer segment phenotypes described in our study. *First*, each PRPH2 mutation analyzed here causes a reduction in the photoreceptor PRPH2 content (Stuck et al., 2014; Zulliger et al., 2018; Chakraborty et al., 2020), which itself could distort outer segment structure (as occurs in the heterozygous *rds* mouse) without affecting the ability of disc membranes to enclose. Related to this point, there are trafficking defects associated with the C213Y mutation (Chakraborty et al., 2020), but not the C150S and Y141C mutations (Stuck et al., 2014; Zulliger et al., 2018), which explains the near absence of outer segment structures in C213Y homozygotes. Immunogold labeling of PRPH2 in C150S and Y141C homozygous mutants showed that each mutant correctly localizes to the disc rim region (Stuck et al., 2014; Zulliger et al., 2018). This suggests that they can incorporate into the disc rim, although it remains to be addressed whether mutant incorporation is complete and whether the final density of mutant PRPH2 molecules is the same as in WT.

Our *second* explanation is that these mutations could affect PRPH2 interactions with ROM1, which has been shown for other PRPH2 mutations (Böhm et al., 2017). Of note, while both ROM1 and PRPH2 can traffic to the outer segment in the absence of one another (Clarke et al., 2000; Lee et al., 2006), trafficking of various PRPH2 mutants (including Y141C) may involve the trafficking of ROM1 (Böhm et al., 2017; Conley et al., 2017). Along this line, outer segments of Y141C mice have been reported to contain ROM1-positive intracellular vesicles (Stuck et al., 2014). Nonetheless, ROM1 knockout only marginally affects outer segment ultrastructure (Clarke et al., 2000), suggesting that altered PRPH2 interactions with ROM1 are unlikely to explain the severity of the observed phenotypes.

Ultimately, the process of disc enclosure remains poorly understood. The only known facet of this process is the distribution of PRPH2 to the rims of disc membranes as they undergo enclosure (Arikawa et al., 1992; Ding et al., 2015; Stuck et al., 2016). While this may suggest that PRPH2 plays an active role in disc enclosure, it is also consistent with this protein simply localizing to the disc rim following the enclosure process, as suggested above. If the latter is the case, there must be other proteins primarily responsible in this membrane remodeling. One potential candidate is prominin-1, a protein shown to be located at the growing edges of newly forming discs (Yang et al., 2008; Han et al., 2012). The knockout of prominin-1 causes a drastic defect in disc formation (Yang et al., 2008; Zacchigna et al., 2009), yet, more work is needed to elucidate the exact role which this protein plays in this complex process. No other strong leads have been identified so far, suggesting that published outer segment proteomes (Liu et al., 2007; Kwok et al., 2008; Reidel et al., 2011; Skiba et al., 2013; Spencer et al., 2019b) may be useful in identifying candidates for future direct testing.

In summary, our study shows that normal PRPH2 oligomerization is not required for disc enclosure. It is still possible that other *PRPH2* mutations do affect enclosure, independently of any effect on oligomerization. Thus, future analyses of other *PRPH2* mutations should include an assessment of disc enclosure to facilitate an understanding of the mechanisms underlying this process and their possible involvement in visual pathology.

## Data Availability Statement

All datasets generated for this study are included in the article.

## Ethics Statement

The animal study was reviewed and approved by Institutional Animal Care and Use Committee University of Houston, Texas, TX, USA.

## Author Contributions

TL, MA-U, VA, and MN designed the experiments. TL, MM, and MK collected eyes. TL processed samples and performed electron microscopy. TL, MA-U, VA, and MN analyzed the images. TL wrote the first draft of the manuscript. All authors read and edited the manuscript.

## Conflict of Interest

The authors declare that the research was conducted in the absence of any commercial or financial relationships that could be construed as a potential conflict of interest.
